# Drug-induced Thrombotic Microangiopathy Caused by Gemcitabine

**DOI:** 10.7759/cureus.3088

**Published:** 2018-08-02

**Authors:** Askari Hasan, Akriti G Jain, Huda Naim, Alvina Munaf, George Everett

**Affiliations:** 1 Florida Hospital, Orlando, USA; 2 Internal Medicine Residency, Florida Hospital, Orlando, USA; 3 Internal Medicine, Dow University of Health Sciences (DUHS), Karachi, PAK; 4 Internal Medicine, Florida Hospital, Orlando, USA

**Keywords:** pancreas cancer, atypical hemolytic uremic syndrome

## Abstract

Hemolytic uremic syndrome (HUS) is the triad of nonimmune (Coombs negative) hemolytic anemia, low platelet count, and renal impairment. HUS has been associated with a variety of gastrointestinal malignancies and chemotherapeutic agents. We present a patient with pancreatic cancer treated with gemcitabine for palliation who developed gemcitabine‐induced HUS (GiHUS) which responded to some extent to blood and platelet transfusions. With the increase in the use of gemcitabine therapy for pancreatic and other malignancies, it is essential to accurately and timely diagnose GiHUS to avoid the life-threatening complications.

## Introduction

Hemolytic uremic syndrome (HUS) is the triad of nonimmune (Coombs negative) hemolytic anemia, low platelet count, and renal impairment [1]. The anemia is severe and microangiopathic in nature, with a platelet count less than 60,000/mm3 in most cases [1].

In children, the disease is most commonly triggered by Shiga-like toxin (Stx)-producing *Escherichia coli*. Non-Shiga toxin-associated HUS can be sporadic or familial and has a poor outcome. Up to 50% of cases progress to end-stage renal disease (ESRD) or have irreversible brain damage, and has an overall 25% mortality [2-4].

HUS has been associated with a variety of gastrointestinal malignancies, most commonly metastatic adenocarcinoma from the stomach, colon, rectum, or pancreas. Certain chemotherapeutic agents have been reported to be associated with HUS, including mitomycin, cisplatin, bleomycin, and more recently gemcitabine [5-6].

## Case presentation

A 66-year-old male with a past medical history of hypertension and pancreatic adenocarcinoma presented to our hospital with complaints of nausea, vomiting, and generalized weakness in the arms and legs. The patient was diagnosed with locally advanced, pancreatic cancer, T1 N0 M0 a year prior to presentation. Magnetic resonance cholangiopancreatography (MRCP) revealed a 1.7 cm mass at the head of his pancreas, locally invasive but without the involvement of lymph nodes, superior mesenteric artery, superior mesenteric vein or portal vein. Endoscopic biopsy revealed adenocarcinoma. The patient was a poor surgical candidate due to social issues, alcoholism, residence at a nursing home and was at a high-risk for post-surgical complications. The patient was treated palliatively with nine cycles of gemcitabine and paclitaxel. The initial dose of gemcitabine was 2000 mg. The tumor decreased in size and CA 19-9 level declined from an initial level of 2000 to 26 units/mL. Later the dose of gemcitabine was reduced to 1400 mg (20% reduction) after the sixth cycle due to pancytopenia.

**Figure 1:**
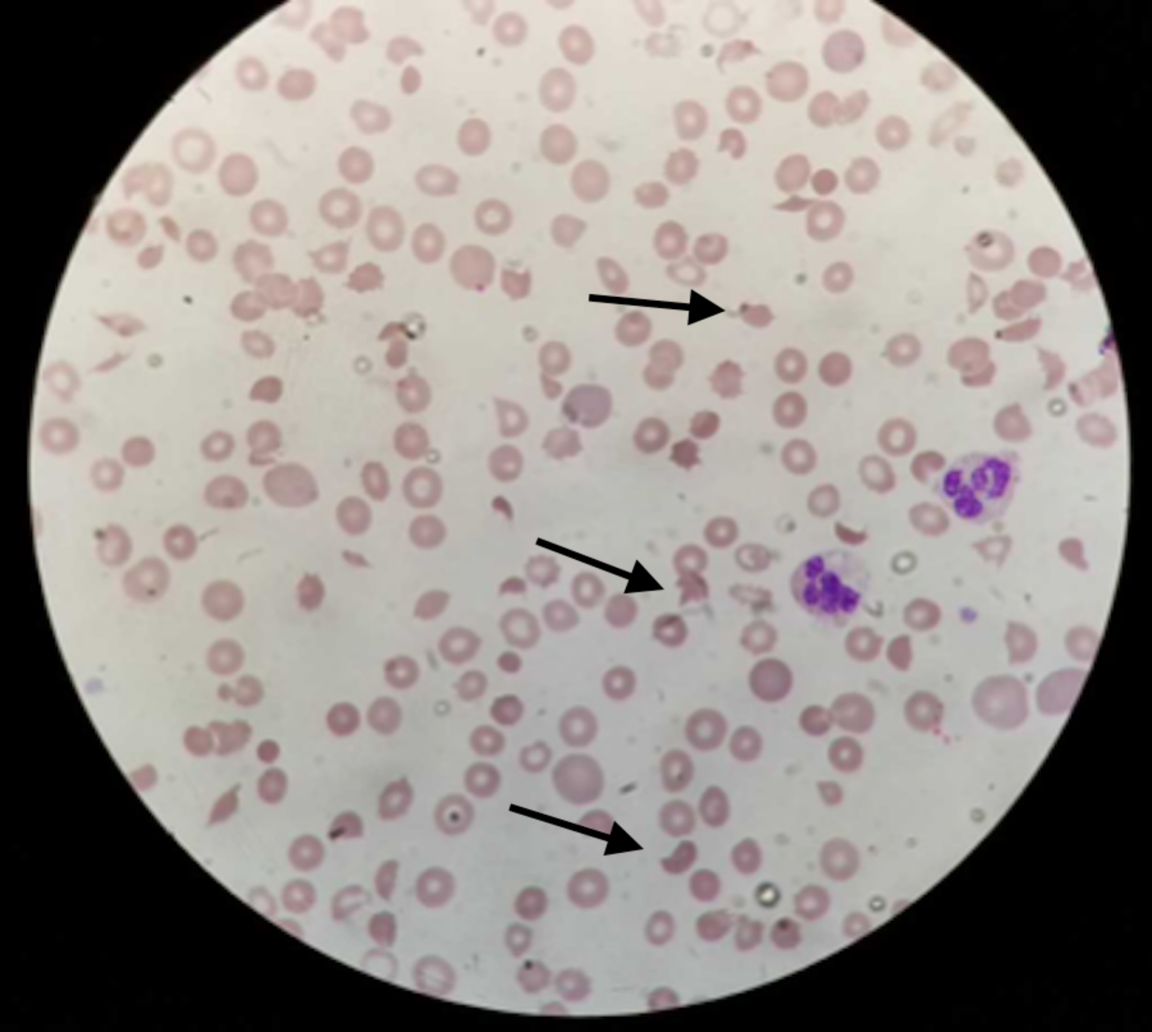
Peripheral blood film showing numerous schistocytes

**Figure 2:**
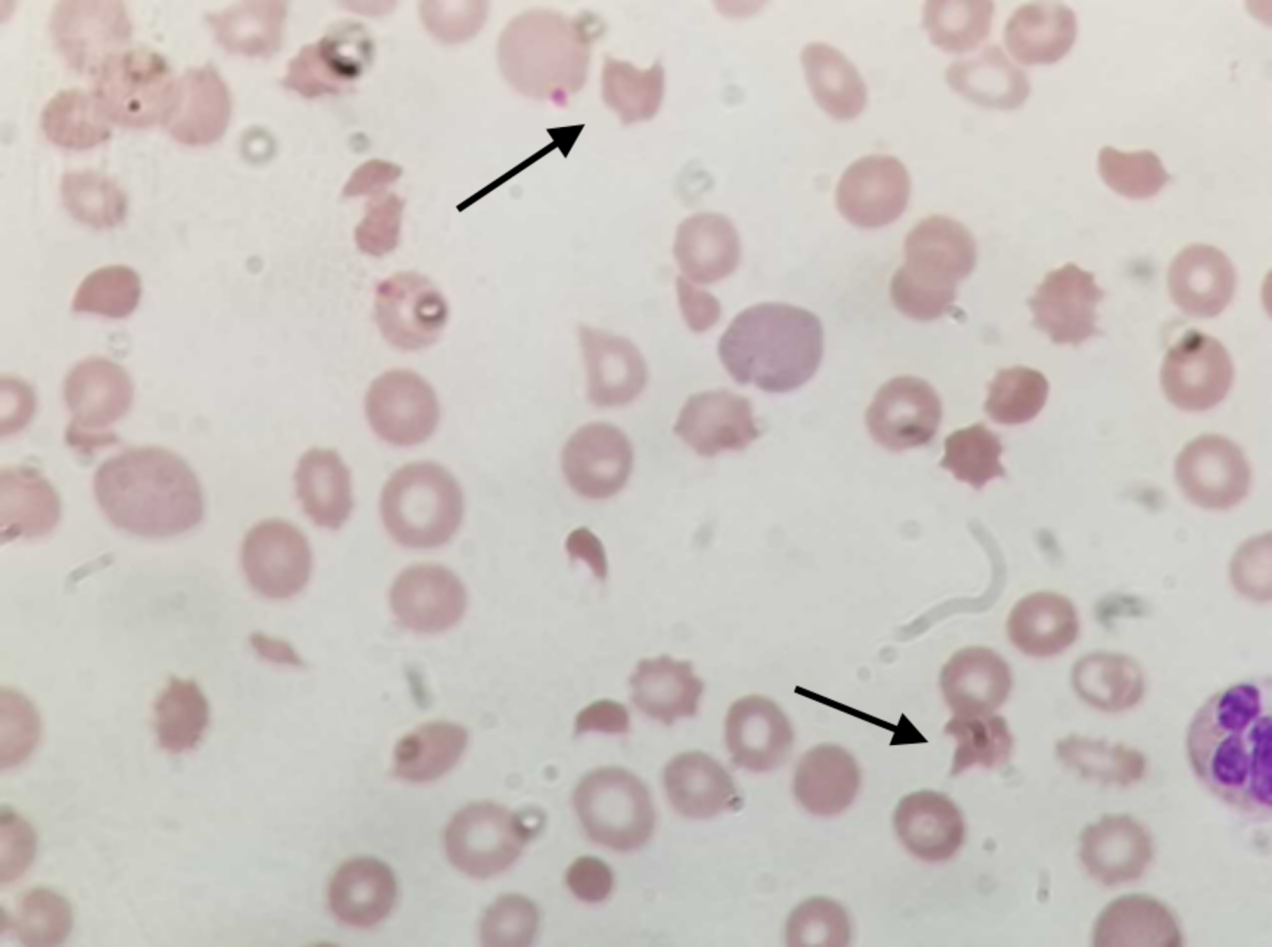
Schistocytes under magnification

**Figure 3:**
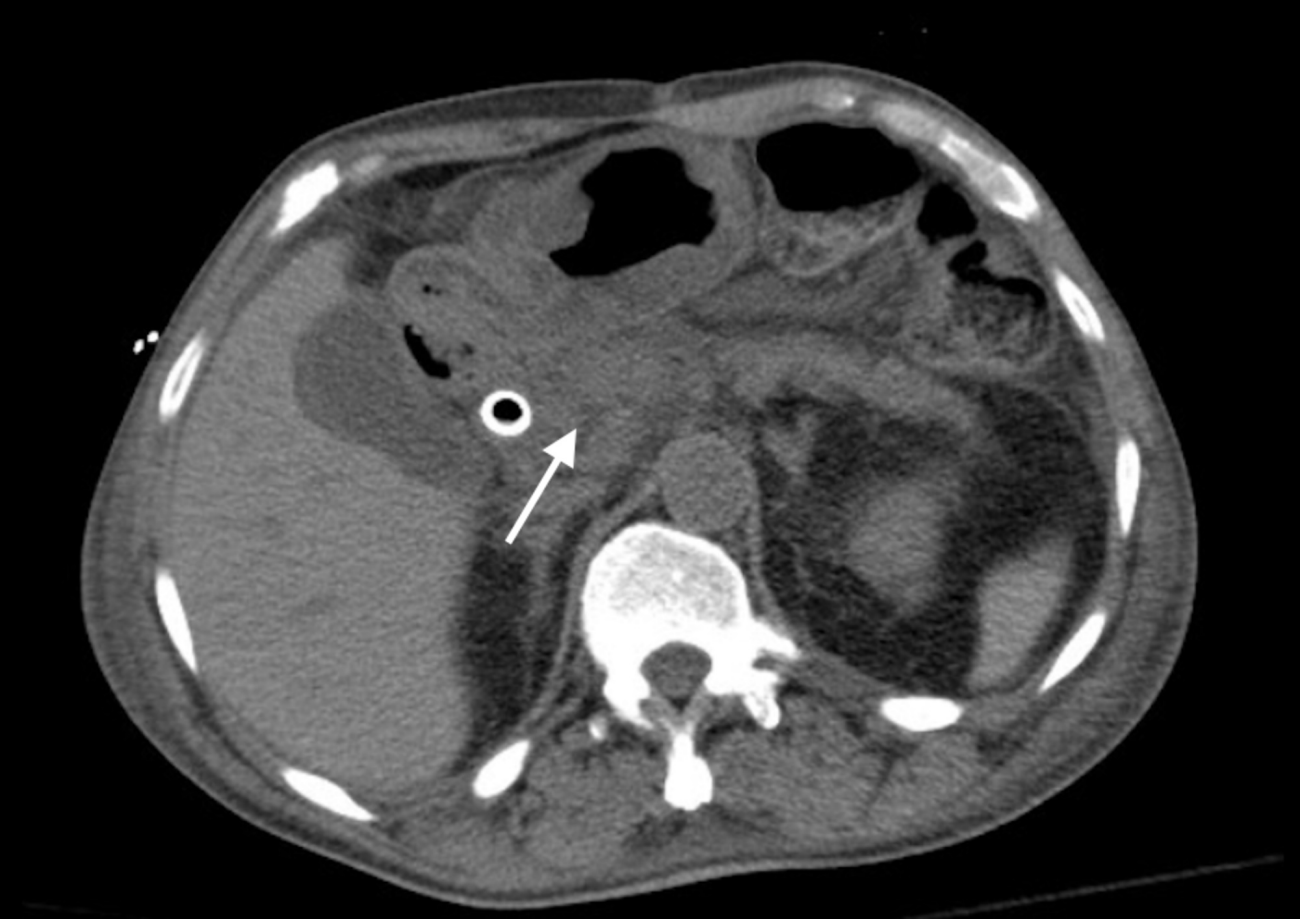
Pancreatic head mass with biliary stent in place

On admission to our hospital, the patient reported abdominal pain that was sharp and located in the right lower quadrant (RLQ). He denied fevers or chills. The patients' vital signs were: temperature 99.3 °F, heart rate of 73 beats per minute, blood pressure 129/60 mmHg, respiratory rate of 17 breaths per minute and oxygen saturation 100% on room air. The physical examination was remarkable for RLQ tenderness. The laboratory data revealed hemoglobin (Hb) 6.5 g/dL, hematocrit (Hct) 19.8, mean corpuscular volume (MCV) 83.2fL /red cell, red cell distribution width (RDW) 19.1 %, white cell count of 9.44 x 109/L, platelets of 54 x 109/L, alanine transaminase (ALT) 133 IU/L, aspartate transaminase (AST) 222 IU/L, alkaline phosphatase (ALP) of 147 IU/L and a total bilirubin of 5 umol/L. BUN was 42 mg/dl, creatinine 2.12 mg/dl (baseline creatinine of 0.8), LDH was 1700 u/l, reticulocyte count was 7.8%. Peripheral smear showed microcytic anemia with frequent schistocytes consistent with a microangiopathic hemolytic process (Figures 1-2). Urinalysis was positive for 1+ blood and 1+ albumin. Computed tomography (CT) scan of the abdomen without contrast showed a stable pancreatic mass and no signs of hydronephrosis (Figure 3). ERCP revealed choledocholithiasis. Choledocholithotomy was performed and subsequently the bilirubin improved. Blood cultures grew Klebsiella. He was treated with piperacillin-tazobactam. The patient received intravenous (IV) fluids, blood and platelets when the Hb and platelets declined to Hb of 6.4 g/dl and platelet counts of 8 x 109. He was treated with methylprednisone 30 mg IV q24 and the platelet count increased to 20 x 109. His creatinine increased to 12 mg/dl and BUN to 112 mg/dl. ADAMTS13 activity was 34%. A diagnosis of GiHUS was made. The patient was offered plasmapheresis, but he opted for hospice.

## Discussion

Gemcitabine is a nucleoside analog structurally related to cytarabine [7] and is indicated for use in multiple malignancies including pancreatic and non-small cell lung cancer, urothelial and ovarian cancers [8]. The incidence of gemcitabine-induced HUS (GiHUS) has been variously reported to be between 0.015% to 4% [9]. The median time between the initiation of chemotherapy and onset of GiHUS was 7.4 months [10].

Clinically it is very difficult to distinguish HUS associated with underlying malignancy from that caused by chemotherapy. There is considerable etiological overlap in biochemical parameters and tumor factors such as tumor necrosis factor-alpha, interleukin-1, and interleukin-6 as well as von Willebrand factor (vWF) antigen and low molecular weight vWF multimers [11-12]. Cancer-associated HUS usually occurs during widespread metastatic disease or poorly controlled carcinomas, whereas chemotherapy-associated HUS is more common when the patient is in disease remission or has minimal tumor burden [13]. Carreras [14] and Hammar [15] suggest initial testing for serum C3 concentrations; however, normal C3 levels do not necessarily exclude a complement dysfunction. More sensitive assays could be a higher-than-normal C3d/C3 ratio in plasma or the presence of C3 deposits in renal biopsy.

GiHUS should be suspected in a patient with malignancy whenever renal dysfunction occurs without an obvious cause. Laboratory investigations suggestive of microangiopathic hemolysis should be obtained including red cell morphology, LDH, fibrin split products and reticulocyte count. A renal biopsy can confirm the diagnosis by revealing the classic microvascular damages with arterioles and small arteries occluded by eosinophilic hyaline thrombi containing fibrin and platelet aggregates [16].

In patients suspected of GiHUS, immediate cessation of gemcitabine is the initial step [17]. GiHUS has since its recognition has been associated with a poor prognosis. After the introduction of plasma manipulation, the mortality rate has decreased from 50% to 25% [18]. Removal of plasma and its subsequent substitution with albumin and saline has not been shown to lead to an increase in the platelet count which was seen in our patient. In patients with renal insufficiency or heart failure, plasma exchange within 24 hours of presentation should be considered as first-choice therapy [1]. In a few patients with extensive microvascular thrombosis at renal biopsy, refractory hypertension, and signs of hypertensive encephalopathy, conventional therapies including plasma manipulation are not enough to control the disease. Bilateral nephrectomy has been performed with favorable results in some patients [19]. Other treatments, including antiplatelet agents, prostacyclin, heparin or fibrinolytic agents, steroids, and intravenous immunoglobulins, have been attempted, with no consistent benefit [1].

Eculizumab, a terminal complement inhibitor, is a humanized monoclonal antibody that binds with high affinity to the human C5 complement protein and blocks the generation of proinflammatory C5a and C5b-9 [20]. Clinical trials report that eculizumab resulted in increases in the platelet count, with a mean increase from baseline to 73 × 109 per liter (P<0.001) by the 26th week. Eculizumab has been associated with continuous, time-dependent increases in the estimated glomerular filtration rate (GFR) and improvement in health-related quality of life.

## Conclusions

With the increase in the use of gemcitabine therapy for pancreatic and other malignancies, it is essential to accurately and timely diagnose GiHUS to avoid the life-threatening complications. Our patient underwent palliative treatment with gemcitabine for pancreatic adenocarcinoma. For any patient receiving gemcitabine like our patient, either therapeutic or palliative, clinicians should have a low threshold for suspecting HUS in the presence of appropriate clinical conditions and laboratory findings especially renal dysfunction.
